# Measuring Positive and Negative Attitudes Associated With the Use of Artificial Intelligence in Nursing Profession: Cross‐Cultural Adaptation and Psychometric Analysis of ASUAITIN

**DOI:** 10.1155/jonm/5888837

**Published:** 2026-04-29

**Authors:** Zhi Chen, Xingxing Wu, Hong Li, Shufen Ke, Rong Lin

**Affiliations:** ^1^ The School of Nursing, Fujian Medical University, No. 1 Xuefu North Road Minhou County, Fuzhou, Fujian, 350122, China, fjmu.edu.cn; ^2^ The School of Nursing, Fudan University, No. 305 Fenglin Road Xuhui District, Shanghai, 200032, China, fudan.edu.cn; ^3^ Nursing Department, The Affiliated Hospital of Putian University, No. 999 Dongzhen East Road Licheng District, Putian, Fujian, 351100, China, ptu.edu.cn

**Keywords:** artificial intelligence, artificial intelligence technologies, attitude, cross-cultural adaptation, nursing, psychometric, reliability, validity

## Abstract

**Background:**

Artificial intelligence (AI) technologies have appeared in many specific clinical nursing scenarios, but its actual adoption effect remains challenging. Assessing nurses’ attitude toward the use of artificial intelligence technologies will help optimize the integration of AI technologies in the field of nursing.

**Aim:**

The aim of this study is to cross‐culturally adapt the Attitude Scale towards the Use of Artificial Intelligence Technologies in Nursing (ASUAITIN) to the Chinese nursing cultural context and verify its reliability and validity in the nurse population.

**Methods:**

This cross‐sectional study adhered to the STROBE guidelines and was designed to translate the ASUAITIN into Chinese. Experts and clinical nurses were invited to discuss and modify the content and concepts, as well as to assess its alignment with the Chinese cultural context. A total of 436 clinical nurses from 20 hospitals in Fujian Province, China, were surveyed from March to April 2025, and the psychometric properties of the Chinese version of ASUAITIN were evaluated.

**Results:**

The Chinese version of ASUAITIN consists of 15 items and 2 dimensions, namely, positive attitude and negative attitude, which demonstrates acceptable content validity. Exploratory factor analysis showed that the scale consists of 2 factors, explaining 68.92% of the total variance, with loadings of each factor ranging from 0.704 to 0.912. Confirmatory factor analysis supported the two‐factor structure and indicated acceptable model fit and good convergent validity and was able to fully represent the scale structure. Meanwhile, the internal consistency and test–retest reliability were satisfactory.

**Conclusion:**

The Chinese version of ASUAITIN showed acceptable validity and reliability and can, therefore, be used to assess Chinese nurses’ attitudes toward the use of AI technologies.

**Implications for Nursing Management:**

The scale provides the nursing management with a practical tool to assess nurses’ attitudes toward the use of AI technologies and promote smoother integration of AI technologies. It supports targeted training programs to enhance nurses’ AI application.

## 1. Introduction

Artificial intelligence (AI) refers to an emerging technical science that studies and develops theories, methods, technologies, and application systems for simulating, extending, and expanding human intelligence. It can perceive the environment through data collection, analyze and interpret information, infer knowledge, reason or process knowledge, obtain information from data, and take the best action to achieve the established goals [1]. It includes machine learning, natural language processing, computer vision, intelligent robots, and automatic programming.

In recent years, AI technologies have rapidly penetrated from theoretical research to clinical practice, and the medical industry, including nursing work, has gradually started the intelligent transformation [2]. In clinical nursing practice, AI technologies have been widely used in specific scenarios such as disease assessment, clinical decision‐making, online education and training platforms for nurses, and reducing the occurrence of adverse events [3–5]. They have not only effectively reduced the rate of human operation errors and improved patient safety and nursing quality [3] but also continued to promote the high‐quality development of the field of nursing [6]. Although AI technologies have shown advantages that cannot be underestimated in the field of nursing, their clinical application still faces many challenges, including the attitude of nurses toward the use of AI technologies, algorithm bias, data privacy risks, and obstacles to data popularization. Therefore, studying the influencing factors during the use of AI technologies is crucial to optimizing the integration of AI technologies in the field of nursing.

Clinical practice shows that the actual adoption rate of AI technologies in the field of nursing is 40%–60% lower than expected, among which nurses’ attitude toward the use of AI technologies is one of the key constraints [7]. Attitude toward the use of AI technologies is defined as a comprehensive reflection of an individual’s emotional tendency and cognitive evaluation of AI technologies, and its performance is two‐sided [8]. In terms of positive attitudes, many studies have confirmed nurses’ recognition of the clinical value of AI technologies. For example, a cross‐sectional study by Xiaoyan Wang et al. showed that many nurses believed that AI technologies could improve the accuracy of nursing diagnosis, improve patient health, and reduce the burden on nursing staff [9]. A survey of nursing students found that most expressed their willingness to apply AI technologies in future clinical practice [10]. In terms of negative attitudes, occupational replacement anxiety is the most prominent, and some nurses are worried that AI technologies may lead to job losses [11]. In addition, some nurses believe that AI technologies are depersonalized and inhumane, lacks human emotions and empathy, and cannot understand and address patients’ emotional needs during the nursing process. This conflicts with the core value of nursing–humanistic care and may lead to a series of ethical issues [12, 13]. Previous studies have pointed out that nurses, as the main providers of health services for patients, have a positive attitude toward AI technologies, which is conducive to the development of AI technologies in the field of nursing [9]. If nurses have a negative attitude toward the use of AI technologies, they will not be able to maximize the benefits of medical AI technologies [14]. Given the key moderating role of attitude on the effectiveness of AI technologies application, systematically evaluating the attitude of nurses towards the application of AI technologies can not only determine the readiness of nurses in medical institutions that will use AI technologies but also provide empirical evidence for the optimization of AI technologies.

Existing measurement tools of attitude towards the use of AI technologies are mainly aimed at the general population or a specific group of medical students, such as the General Attitudes Toward Artificial Intelligence Scale (GAAIS) developed by Schepman and Rodway for adults over 18 years old in the United Kingdom [15, 16]. The Attitudes Toward Artificial Intelligence Scale designed by Dos Santos et al. is aimed at medical students, but it only focuses on the field of radiology and lacks applicability in nursing scenarios [17]. In contrast, there is a lack of Chinese version of the evaluation tool for nurses’ attitude of AI technologies that takes into account the professional characteristics of nurses and has undergone cross‐cultural adaptation. Based on this, exploring a Chinese version of the attitude of AI technologies scale for nurses with good reliability and validity has become a prerequisite for promoting the deep integration of AI technologies and nursing practice. A review shows that the Attitude Scale toward the Use of Artificial Intelligence Technologies in Nursing (ASUAITIN) developed by Yılmaz et al. is the first standardized measurement tool specifically for nurses [18]. The scale includes two dimensions: positive and negative attitudes and can comprehensively evaluate nurses’ cognitive tendencies towards AI technologies. According to the technology acceptance model (TAM), an individual’s behavioral intention to adopt new technologies depends primarily on two cognitive beliefs: perceived usefulness and perceived ease of use. These beliefs shape positive attitudes toward technology use. Conversely, negative attitudes may stem from perceived risk, ethical concerns, or threats to professional autonomy—factors not explicitly reflected in the original TAM but increasingly valued in health technology acceptance research [19, 20]. Therefore, the ASUAITIN model, with its two‐factor structure of positive and negative attitudes, can operationally represent the facilitators and inhibitors of AI acceptance in the nursing field. The English version of ASUAITIN has shown satisfactory validity and reliability in a survey of 200 nurses in clinical fields in English [18]. In the field of nursing in China, professional values are deeply influenced by Confucian cultural traditions, emphasizing collectivism, harmony, risk aversion, and humanistic care [21]. These cultural characteristics may simultaneously contribute to positive attitudes toward AI as a tool to improve team efficiency and patient safety, as well as negative attitudes stemming from concerns about dehumanization, ethical conflicts, and technological uncertainty. Importantly, these two dimensions of attitude are not two ends of the same continuum, but rather coexisting and independent structures—a pattern consistent with the dialectical thinking prevalent in East Asian cultures [22]. Based on this, considering the cultural specificity of Chinese nursing practice, the scale should be cross‐culturally adapted and validated for nurses from Chinese cultural background so that the scale can provide meaningful and valid results in the assessment process.

Therefore, this study aims to translate the ASUAITIN into Chinese, conduct cross‐cultural adaption, and conduct psychometric validation among Chinese nurses in order to establish an assessment tool of attitude towards AI technologies suitable for Chinese nurses. The application of this tool will help accurately assess the attitude characteristics of nurses toward AI technologies, provide evidence‐based basis for medical institutions to develop targeted training programs of AI technologies, promote the organic integration of nursing practice and AI technologies, and thus promote the high‐quality development of nursing services toward intelligence and precision.

## 2. Methods

### 2.1. Aim

This study aims to cross‐culturally adapt ASUAITIN to the Chinese nurse population and verify its validity and reliability.

### 2.2. Design and Participants

**FIGURE 1:**
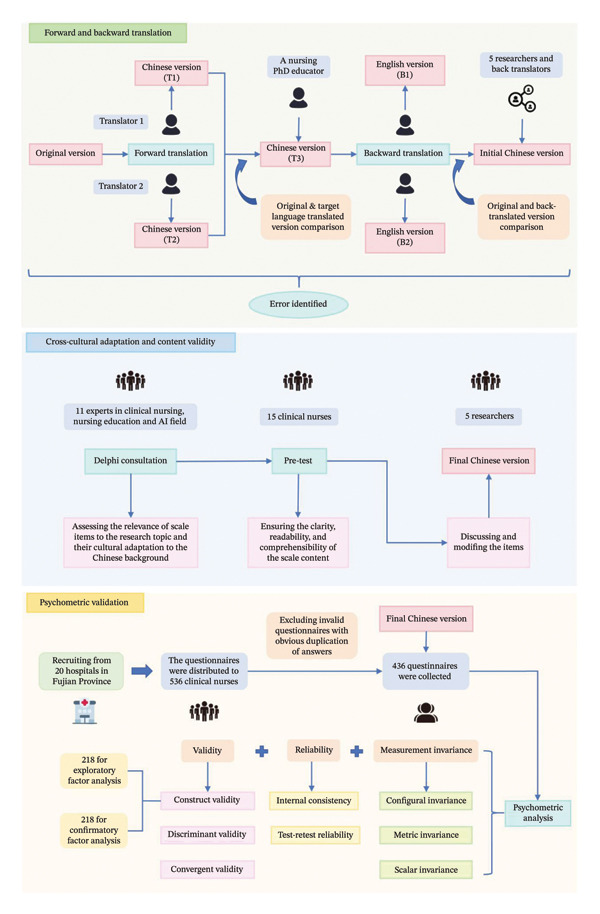
The translation, cultural adaptation process, and psychometric validation process.

This study was a cross‐sectional psychometric study using a three‐stage structured process and adhered to the Strengthened Reporting of Observational Studies in Epidemiology (STROBE) guidelines for reporting observational studies. In the first stage, ASUAITIN was translated from English into Chinese using forward and backward translation. In the second stage, experts were invited to conduct cultural adaptation and content evaluation, and a pretest was conducted in a small group of nurses. In the third stage, the Chinese version of ASUAITIN was validated through a cross‐sectional survey of Chinese clinical nurses. Convenience sampling was used to recruit participants from 20 hospitals in Fujian Province, China. Inclusion criteria are as follows: (a) engaged in clinical nursing for 1 year or more and (b) voluntary participation in this study. Exclusion criteria are as follows: (a) nurses working in nonclinical departments and (b) nurses not working in the hospital during the survey period (nurses currently retired or on leave due to illness or personal reasons). The translation, cultural adaptation process and psychometric validation process is shown in Figure 1 **.**

### 2.3. Phase I: Instrument Translation

#### 2.3.1. English Version of ASUAITIN

The English version of ASUAITIN was developed by Yılmaz et al. [18] and is a tool for assessing clinical nurses’ attitudes toward the practice of AI technologies. The scale includes two dimensions and 15 items: nurses’ positive attitudes toward AI technologies in nursing practice (9 items) and negative attitudes (6 items). For example, “I think that the nursing profession will be harmed if AI technologies are used more in the future” is negative, and “There are many beneficial applications of AI technologies in nursing” is positive. Each item is assessed on a five‐way Likert‐type scale of 1–5, with high scores indicating a positive attitude. The highest score obtainable on the scale is 75, and the lowest is 5. For details of the scale, please see the Supporting Information 1. In the original study, the loadings of all items in the scale ranged from 0.529 to 0.866. The Cronbach’s α coefficient of the total scale is 0.910, the Cronbach’s α coefficient of factor 1 is 0.933, and the Cronbach’s α coefficient of factor 2 is 0.917 [18].

#### 2.3.2. Forward and Back Translation

After obtaining the authorization and consent of the original scale author via email, the Brislin translation model was used for translation [23]. Initially, we performed forward translation: ASUAITIN was independently translated from English into Chinese versions T1 and T2 by two professionals familiar with both languages and local culture. Next, a nursing PhD educator was invited to compare the differences between the translated versions and the original ASUAITIN, and the Chinese versions T1 and T2 were merged into one version, namely, T12. Then, we performed reverse translation: two native English translators who were not familiar with the original scale were invited to translate T12 into English versions (B1 and B2). Finally, the researchers and the back translators compared the original English ASUAITIN and the backtranslated English ASUAITIN and discussed the accuracy of the translation and the readability of the scale language. Combined with the Chinese context, the first draft of the Chinese version of ASUAITIN was finally formed.

### 2.4. Phase II: Cross‐Cultural Adaptation and Content Validation

Through Delphi consultation and clinical nurses’ feedback, determine whether the scale items need to be modified to adapt to the Chinese nursing context.

#### 2.4.1. Delphi Consultation

To ensure the professional rigor and cultural appropriateness of the scale, an expert panel of 11 members was invited to independently review the Chinese version of the ASUAITIN. Each expert, drawing on their professional knowledge and clinical experience, evaluated the content and concepts of each item in the scale, assessed its suitability for the Chinese cultural context, and provided suggestions for modification [24]. The expert group’s research areas cover clinical nursing, nursing education, nursing informatics, and nursing management. Among them, 6 experts hold doctoral degrees and 5 hold master’s degrees. The average age of the panel was 38.36 years (standard deviation = 8.62), and the average work experience was 14.27 years (standard deviation = 8.53). Through the adoption of a self‐evaluation method, the authority coefficient (Cr) was determined by considering the familiarity coefficient (Cs) and the judgment coefficient (Ca). The formula for calculating the Cr is Cr = (Cs + Ca)/2 [25]. The expert authority coefficient was 0.823. For details of the Delphi consultation, please see the Supporting Information 2. The relevance of the scale items to the research topic was determined by the Likert 4‐level scoring method, with 1–4 points from “irrelevant” to “very relevant.” The content validity index at the item level (I‐CVI) and the average content validity index at the scale level (S‐CVI/Ave) were calculated based on the scoring results. When I‐CVI ≥ 0.78 and S‐CVI/Ave > 0.9, it indicates that the scale has good content validity [26].

#### 2.4.2. Pre‐test

To ensure the clarity, readability, and comprehensibility of the scale content, we selected 15 clinical nurses from Fujian Provincial Hospital for pre‐test through convenience sampling based on the recommendation of a sample size of 10–40 participants [27] and used the online questionnaire survey platform “Wenjuanxing” (https://www.wjx.cn/) to collect data. The 15 nurses were asked to carefully evaluate the clarity of the scale and mark unclear items. If there were unclear items, the researchers were required to discuss and modify the items.

### 2.5. Phase III: Psychometric Validation

The psychometric properties of the Chinese version of ASUAITIN include its construct validity, discriminant validity, convergent validity, internal consistency, test–retest reliability, and measurement invariance.

#### 2.5.1. Sample and Data Collection

Participants were recruited from 20 hospitals in Fujian Province, China, using a convenience sampling method from March to April 2025. The sample size was determined based on three recommendations: (a) 5–10 participants per item to accurately evaluate construct validity and internal consistency [28]; (b) using different subsamples to conduct exploratory factor analysis (EFA) and confirmatory factor analysis (CFA) to facilitate a cross‐validation test [29]; and (c) a minimum of 200 participants per subsample for EFA and CFA [30, 31]. Following these three recommendations, a minimum sample size of 400 participants was considered adequate for our study. Assuming 10% of possible missing responses, we planned to recruit at least 440 participants.

This study used electronic questionnaires to collect data. After obtaining the consent of the director of the hospital’s nursing department, electronic QR codes containing the informed consent form and the questionnaire link were distributed to participants. The homepage of the questionnaire detailed the purpose, significance, and questionnaire filling method of the study. Participants answered the questions voluntarily and anonymously. To ensure the quality of the questionnaire and reduce duplicate submissions, all questions were set as mandatory answers and the survey platform applied device level submission restrictions. A total of 536 questionnaires were distributed, and after excluding invalid questionnaires with obvious duplication of answers, a total of 436 questionnaires were collected, with an effective recovery rate of 81.34%.

#### 2.5.2. General Information Questionnaire

This questionnaire was designed by the researchers based on previous studies to collect the participants’ sociodemographic characteristics and their knowledge and application of AI technologies, including gender, age, years of work experience, education level, marital status, job title, hospital department, weekly working hours, degree of understanding of the application of AI technologies in healthcare and nursing, personal understanding and proficiency of the use of AI technologies, the current status of AI technologies introduction in the institution, and the frequency of personal use of AI technologies. Only age and years of work experience were treated as continuous variables because participants could provide this information directly and efficiently. The remaining variables were categorized to simplify questionnaire completion and improve response feasibility. For details of the questionnaire, please see the Supporting Information 1.

#### 2.5.3. Data Analysis

SPSS 26.0 and AMOS 24.0 software were used to perform descriptive statistical analysis and reliability and validity tests on the data. Measurement data were expressed as mean ± standard deviation (Mean ± SD), and count data were expressed as frequency and percentage. *p* < 0.05 was considered statistically significant. The 436 data were randomly divided into two subsamples using the SPSS random number table function. Subsample 1 was used for item analysis and EFA (*n* = 218), and Subsample 2 was used for CFA and reliability and validity tests (*n* = 218) [30].

##### 2.5.3.1. Item Analysis

Item analysis was performed using the corrected item‐total correlation (CITC) and critical ratio method. (a) Correlation analysis was performed between the scores of each item and the total score in the scale. When *r* > 0.4, it indicated that the item was highly correlated with the total scale and could be retained. (b) The 218 datasets were sorted from high to low according to the total score and divided into a high score group (top 27%) and a low score group (bottom 27%). The *T* value was calculated using a two‐sample independent *t*‐test. Critical ratio higher than 3 indicated that the item had good discrimination and could be retained [32].

##### 2.5.3.2. Structural Validity

The structural validity included EFA and CFA [33]. We used Kaiser–Meyer–Olkin (KMO) test and Bartlett’s test of sphericity to verify the applicability of factor analysis [34]. When KMO > 0.70 and *p* < 0.05, the data were considered suitable for factor analysis. EFA used the maximum variance orthogonal rotation method to perform principal component analysis (PCA). The appropriate standard for extracting factors is that the eigenvalue is equal to or greater than 1.00. When the factor loading of each item is greater than 0.4 and the cumulative variance contribution rate is greater than 50%, it indicates that the structural validity of the scale is good [35]. CFA uses the maximum likelihood method to test the factor structure obtained by EFA. When relative chi‐square/degree of freedom (*χ* ^2^ */*df) < 3.00, root mean square error of approximation (RMSEA) and standardized root mean square residual (SRMR) are both < 0.10 [36], and comparative fit index (CFI) and Tucker–Lewis goodness‐of‐fit index (TLI) are both > 0.90, it indicates that the scale has a good fit [37].

##### 2.5.3.3. Convergent Validity

The average variance extracted (AVE) and composite reliability were used for evaluation. When AVE > 0.50 and composite reliability > 0.70, it indicated that the convergent validity was good [38].

##### 2.5.3.4. Internal Consistency and Test–Retest Reliability

The Cronbach’s α coefficient and test–retest reliability were used for evaluation. When the Cronbach’s α coefficient of the scale was higher 0.7, it indicated that the internal consistency of the scale was good [39]. After obtaining the voluntary consent of the participants, we utilized an online survey platform to generate a set of anonymous system codes; researchers were unable to derive any identifiable address information from these codes, thereby fully safeguarding the participants’ anonymity. Ultimately, we selected 50 participants who completed the questionnaire again two weeks after the initial assessment to obtain test–retest reliability. When test–retest reliability > 0.70, it indicated that the stability of the scale was good [40].

##### 2.5.3.5. Measurement Invariance

Measurement invariance was examined. First, configural invariance was tested by evaluating whether the same factor structure held across both groups. Then, metric invariance was examined by constraining factor loadings to be equal between groups, ensuring that constructs were measured similarly. Finally, scalar invariance was assessed by additionally constraining intercepts to test whether group differences in observed scores reflected latent trait differences rather than measurement bias [41–44]. Model fit was assessed by changes in the CFI, TLI, RMSEA, and SRMR, as the chi‐square nonsignificance criterion is often difficult to achieve in large samples. Cutoff criteria of ≤ 0.010 for CFI and TLI change, ≤ 0.015, and cutoff criteria of for RMSEA change were applied to determine each level of measurement invariance [41, 44].

### 2.6. Ethical Consideration

This study was conducted in strict accordance with the ethical guidelines of the Declaration of Helsinki [45] and has been approved by the Biomedical Research Ethics Review Committee of Fujian Medical University (approval number: 2025–307). All participants voluntarily participated in this study with informed consent, and the research process was conducted anonymously. The research team promised that all data would only be used for the purpose of this study and ensured that participants had the right to withdraw from the study at any time without any adverse effects.

## 3. Results

### 3.1. Characteristics of the Participants

A total of 436 nurses participated in this study, most of whom were female (*n* = 408, 93.58%). A total of 235 (53.90%) were aged from 31 to 40 years old, and 132 (30.28%) had 11–15 years of work experience. A total of 335 (76.83%) of the nurses had Bachelor’s degree or above, and most of them had the titles of senior nurse and senior nurse in charge (*n* = 323, 74.08%). A total of 251 (57.57%) of the nurses worked less than 41 h per week. A total of 246 (56.42%) of the nurses had an average understanding of AI technologies, 189 (43.35%) of the nurses occasionally (monthly/yearly use) used AI technologies, and only 8 (1.83%) of the nurses believed that their proficiency of the use of AI technologies reached an excellent level. Demographics and characteristics of 436 participants are listed in Table 1.

**TABLE 1:** Demographics and characteristics of participants (total *N* = 436).

	**Total sample (*n* = 436)**	**EFA sample (*n* = 218)**	**CFA sample (*n* = 218)**
**Variable**	***N* (%)**		

Gender			
Male	28 (6.42%)	10 (4.59%)	18 (8.26%)
Female	408 (93.58%)	208 (95.41%)	200 (91.74%)
Age, years			
Less than 31 years old	132 (30.28%)	58 (26.61%)	74 (33.94%)
31–40 years old	235 (53.90%)	114 (52.29%)	121 (55.50%)
More than 40 years old	69 (15.83%)	46 (21.10%)	23 (10.55%)
Years in nursing			
Less than 6 years	88 (20.18%)	34 (15.60%)	54 (24.77%)
6–10 years	119 (27.29%)	65 (29.82%)	54 (24.77%)
11–15 years	132 (30.28%)	61 (27.98%)	71 (32.57%)
More than 15 years	97 (22.25%)	58 (26.61%)	39 (17.89%)
Educational level			
College degree and below	101 (23.17%)	52 (23.85%)	49 (22.48%)
Bachelor’s degree	308 (70.64%)	154 (70.64%)	154 (70.64%)
Master’s degree and above	27 (6.19%)	12 (5.50%)	15 (6.88%)
Marital status			
Single	105 (24.08%)	51 (23.39%)	54 (24.77%)
Married	314 (72.02%)	158 (72.48%)	156 (71.56%)
Other^a^	17 (3.90%)	9 (4.13%)	8 (3.67%)
Job title			
Nurse	78 (17.89%)	35 (16.06%)	43 (19.72%)
Senior nurse	143 (32.80%)	65 (29.82%)	78 (35.78%)
Senior nurse in charge	180 (41.28%)	99 (45.41%)	81 (37.16%)
Associate chief senior nurse and above	35 (8.03%)	19 (8.72%)	16 (7.34%)
Hospital department			
Internal medicine	127 (29.13%)	74 (33.94%)	53 (24.31%)
Surgery	86 (19.72%)	35 (16.06%)	51 (23.39%)
Emergency	54 (12.39%)	29 (13.30%)	25 (11.47%)
Intensive care units	25 (5.73%)	11 (5.05%)	14 (6.42%)
Other^b^	144 (33.03%)	69 (31.65%)	75 (34.40%)
Weekly working hours			
Less than 40 h	251 (57.57%)	120 (55.05%)	131 (60.09%)
More than 39 h	185 (42.43%)	98 (44.95%)	87 (39.91%)
Awareness of the use of AI in healthcare			
Yes	219 (50.23%)	113 (51.83%)	106 (48.62%)
No	217 (49.77%)	105 (48.17%)	112 (51.38%)
Awareness of the use of AI in nursing			
Yes	209 (47.94%)	113 (51.83%)	114 (52.29%)
No	227 (52.06%)	105 (48.17%)	104 (47.71%)
Understanding of AI technologies			
Poor	12 (2.75%)	5 (2.29%)	7 (3.21%)
Fair	246 (56.42%)	121 (55.50%)	125 (57.34%)
Good	128 (29.36%)	64 (29.36%)	64 (29.36%)
Very good	37 (8.49%)	20 (9.17%)	17 (7.80%)
Excellent	13 (2.98%)	8 (3.67%)	5 (2.29%)
Frequency of the use of AI technologies			
Frequent (daily use)	37 (8.49%)	27 (12.39%)	10 (4.59%)
Regular (weekly use)	123 (28.21%)	53 (24.31%)	70 (32.11%)
Occasional (monthly/yearly use)	189 (43.35%)	96 (44.04%)	93 (42.66%)
Never	87 (19.95%)	42 (19.27%)	45 (20.64%)
Proficiency of the use of AI technologies			
Poor	67 (15.37%)	35 (16.06%)	32 (14.68%)
Below average	162 (37.16%)	68 (31.19%)	94 (43.12%)
Average	159 (36.47%)	91 (41.74%)	68 (31.19%)
Above average	40 (9.17%)	21 (9.63%)	19 (8.72%)
Excellent	8 (1.83%)	3 (1.38%)	5 (2.29%)
Adoption of AI technologies in the hospital			
Yes	291 (66.74%)	154 (70.64%)	137 (62.84%)
No	145 (33.26%)	64 (29.36%)	81 (37.16%)

*Note: N*, sample size.

Abbreviations: CFA, confirmatory factor analysis; EFA, exploratory factor analysis.

^a^Other category include separated, divorced, remarried, and widowed.

^b^Other category include gynecology, pediatrics, health management, and more.

### 3.2. Cross‐Cultural Adaptation Results of the ASUAITIN

Based on Delphi consultations with 11 experts, we carefully reviewed and discussed the Chinese version of the ASUAITIN and made minor adjustments to some items, while keeping the total number of items unchanged, to improve its comprehensibility and contextual appropriateness. The revisions based on expert consultations are as follows: Item 1 was changed from “I think AI technologies will be a hindrance to the application of nursing care practices” to “I think AI technologies are detrimental to the development of nursing work”, making the tone milder and more consistent with the scale’s purpose of measuring attitudes rather than making definitive predictions; Item 3 was changed from “I think that the nursing profession will be harmed if artificial intelligence technologies are used more in the future” to “I think increased use of artificial intelligence technologies in the future will lead to harm to the nursing profession,” improving the sentence structure for a more fluent and formal written expression; Item 4 was changed from “I think using AI technology in nursing will endanger patient safety” to “I think using AI technology in nursing services will put patient safety at risk,” a more formal and cautious statement that better reflects the tone of an attitude scale; Item 13 was changed from “I would like to have skills in learning and using AI technologies in nursing” to “I would like to have skills in learning and using AI technologies in the field of nursing,” adding “in the field of nursing” to clarify the context of learning and application. In addition, we made several wording changes to other items to enhance their cultural relevance and clarity, reflecting more nuanced and professional phrasing in the Chinese healthcare context. In the pretest conducted with 15 clinical nurses, participants indicated that the items were clear and effectively captured the intended constructs, thus requiring no substantial modifications.

### 3.3. Item Analysis

In the comparison between the high score group and the low score group of the Chinese version of ASUAITIN, critical ratio of all items ranged from 9.757 to 20.934, all of which were higher than 3, so no item needed to be eliminated. In the homogeneity test, the correlation coefficients of all items with the total score ranged from 0.608 to 0.865, all of which were higher than 0.40. The results showed that all items met the requirements and no item needed to be eliminated, see Table 2 **.**

**TABLE 2:** Item analysis (*N* = 218).

Items	Mean ± SD	*r*	CR
ASUAITIN1	3.78 ± 1.12	0.608	13.056
ASUAITIN2	3.81 ± 1.01	0.839	20.682
ASUAITIN3	3.70 ± 1.04	0.865	20.934
ASUAITIN4	3.49 ± 0.96	0.81	15.405
ASUAITIN5	3.77 ± 0.98	0.856	18.711
ASUAITIN6	3.32 ± 1.01	0.668	12.053
ASUAITIN7	3.62 ± 0.86	0.640	10.873
ASUAITIN8	3.62 ± 0.86	0.674	10.800
ASUAITIN9	3.82 ± 0.79	0.803	12.554
ASUAITIN10	4.02 ± 0.85	0.732	11.468
ASUAITIN11	3.99 ± 0.79	0.798	12.080
ASUAITIN12	3.98 ± 0.76	0.851	13.509
ASUAITIN13	4.06 ± 0.77	0.829	12.591
ASUAITIN14	4.04 ± 0.78	0.772	9.757
ASUAITIN15	3.86 ± 0.85	0.680	10.261

*Note: r*, corrected item‐total correlation,.

Abbreviations: CR, critical ratio; SD, standard deviation.

### 3.4. Validity

#### 3.4.1. Content Validity

The I‐CVI of the Chinese version of ASUAITIN at the item level was 0.909–1.000, all above 0.78, and the S‐CVI/Ave at the scale level was 0.994, higher than 0.90. These results show that the Chinese version of ASUAITIN has good content validity.

#### 3.4.2. Construct Validity

**FIGURE 2:**
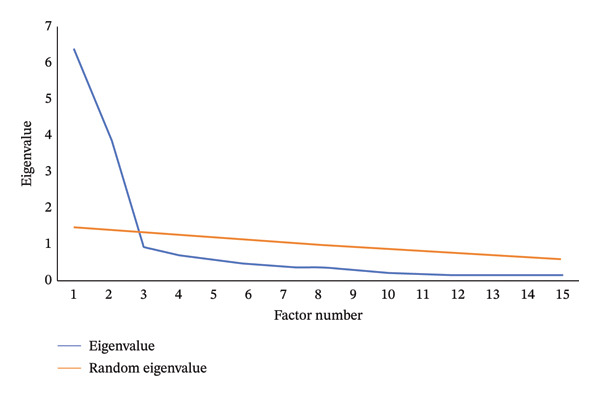
A scree plot for the number of factors to be retained in the exploratory factor analysis.

**FIGURE 3:**
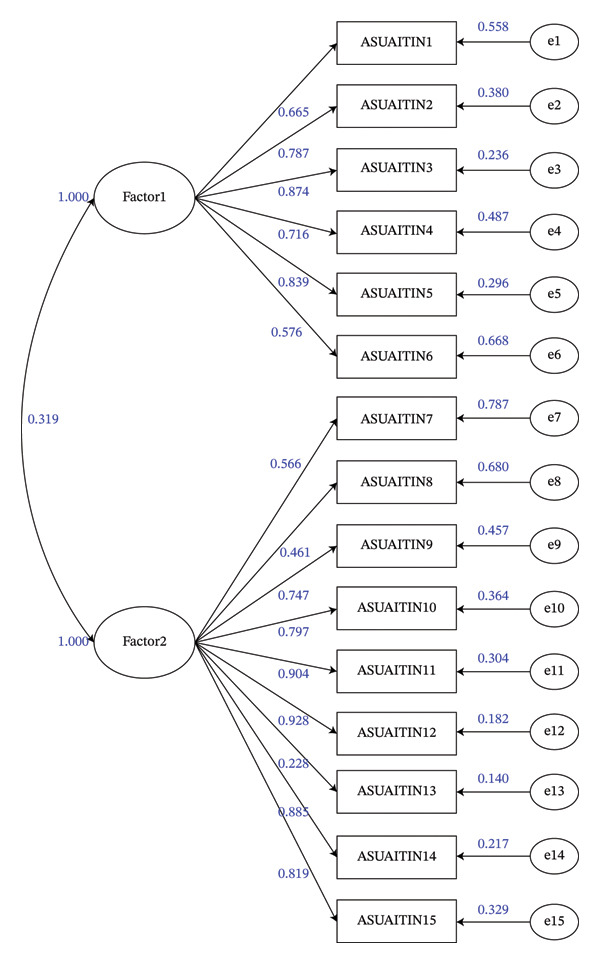
CFA diagram. Factor loadings are provided on the plot. Factor 1, negative attitude; Factor 2, positive attitude.

The evaluation of the construct validity of the scale was conducted in two phases: EFA and CFA. The results of EFA showed that the KMO of the Chinese version of ASUAITIN was 0.886, and the Bartlett sphericity test *χ* ^2^ = 2708.135 (*p* < 0.001), indicating that the data are suitable for factor analysis. The principal component method was used for analysis, and the maximum variance method was used for rotation. A total of two factors with principal components greater than 1 were extracted. The results of parallel analysis showed that the eigenvalue of the data in this study was lower than the simulated eigenvalue when there were three factors, so it was suitable to extract two factors (see Figure 2). The cumulative variance explanation rate of the two factors was 68.92%, and the load of each factor was 0.704–0.912. Factor 1 (negative attitude) included 6 items from ASUAITIN 1 to ASUAITIN 6, with a variance explanation rate of 42.44%. Factor 2 (positive attitude) included 9 items from ASUAITIN 7 to ASUAITIN 15, with a variance explanation rate of 26.49% (see Table 3). The above shows that the structural validity of the scale is good. The CFA diagram is shown in Figure 3.

**TABLE 3:** Rotated component matrix in EFA (*N* = 218).

Items	Factor 1	Factor 2
ASUAITIN1	0.037	**0.704**
ASUAITIN2	0.125	**0.887**
ASUAITIN3	0.078	**0.912**
ASUAITIN4	0.015	**0.880**
ASUAITIN5	0.172	**0.901**
ASUAITIN6	0.003	**0.771**
ASUAITIN7	**0.717**	−0.077
ASUAITIN8	**0.748**	−0.080
ASUAITIN9	**0.844**	0.072
ASUAITIN10	**0.792**	0.095
ASUAITIN11	**0.845**	0.093
ASUAITIN12	**0.885**	0.145
ASUAITIN13	**0.868**	0.143
ASUAITIN14	**0.823**	0.090
ASUAITIN15	**0.738**	0.152
Eigenvalues	6.365	3.973
Variance explained (%)	42.44%	26.49%
Total variance explained (%)	42.44%	68.92%

*Note:* Factor 1, negative attitude; Factor 2, positive attitude. The bold values indicate the primary factor loading for each item.

The results of CFA showed that *χ* ^2^ */*df = 2.752, RMSEA = 0.090, CFI = 0.940, TLI = 0.925, SRMR = 0.055, and the fit indices indicated acceptable, though not optimal, model fit. Further model comparison found that the single‐factor model did not fit acceptable, while the two‐factor model fit acceptable, indicating that the scale has good discriminant validity. For details, see Tables 4 and 5.

**TABLE 4:** Confirmatory factor analysis (*N* = 218).

Dimensions	Items	*b*	*β*	SE	*T*	*p*
Factor 1	ASUAITIN1	1.000	0.665	—	—	—
	ASUAITIN2	1.103	0.787	0.109	10.160	< 0.001
	ASUAITIN3	1.163	0.874	0.109	10.673	< 0.001
	ASUAITIN4	1.000	0.716	0.108	9.298	< 0.001
	ASUAITIN5	1.044	0.839	0.099	10.575	< 0.001
	ASUAITIN6	0.762	0.576	0.100	7.660	< 0.001

Factor 2	ASUAITIN7	1.000	0.461	—	—	—
	ASUAITIN8	1.288	0.566	0.155	8.294	< 0.001
	ASUAITIN9	1.509	0.737	0.191	7.908	< 0.001
	ASUAITIN10	1.668	0.797	0.238	7.000	< 0.001
	ASUAITIN11	1.577	0.834	0.223	7.085	< 0.001
	ASUAITIN12	1.682	0.904	0.232	7.251	< 0.001
	ASUAITIN13	1.666	0.928	0.228	7.306	< 0.001
	ASUAITIN14	1.658	0.885	0.230	7.210	< 0.001
	ASUAITIN15	1.712	0.819	0.243	7.040	< 0.001

*Note:* Factor 1, negative attitude; Factor 2, positive attitude.

**TABLE 5:** Discrimination validity test of the scale (*N* = 218).

Model	*χ* ^2^ */*df	RMSEA (CI 90%)	CFI	TLI	SRMR
Single factor	8.811	0.189 (0.177–0.202)	0.727	0.667	0.169
Two factor	2.752	0.090 (0.076–0.103)	0.940	0.925	0.055

#### 3.4.3. Convergent Validity

The AVE of the two dimensions of the Chinese version of ASUAITIN are 0.562 and 0.615, and the composite reliability are 0.883 and 0.933, indicating that the Chinese version of ASUAITIN has good convergent validity and composite reliability.

### 3.5. Reliability Analysis

The Cronbach’s α coefficient of the Chinese version of ASUAITIN is 0.890, and the Cronbach’s α coefficients of each dimension are 0.885 and 0.933, respectively. The McDonald’s omega coefficients of each dimension are 0.920 and 0.931, respectively. The retest reliability of the total scale is 0.924, and the retest reliability of each dimension is 0.864 and 0.937 (see Table 6), indicating that the Chinese version of ASUAITIN has good internal consistency and stability.

**TABLE 6:** Internal consistency and test–retest reliability (*N* = 218).

Dimensions	Number of items	Cronbach’s α coefficient	Test–retest reliability
Factor 1	6	0.885	0.864
Factor 2	9	0.933	0.937
Total scale	15	0.890	0.924

### 3.6. Measurement Invariance of the Chinese Version of ASUAITIN

To examine whether the factor structure of the Chinese version of ASUAITIN remains stable across subgroups with different levels of AI‐related exposure and competency, we conducted multigroup CFA to test measurement invariance. Three grouping variables were defined based on participants’ self‐reported responses from the demographic section: (1) awareness of AI in healthcare (Yes vs. No), (2) awareness of AI in nursing practice (Yes vs. No), and (3) adoption of AI technologies in the hospital (Yes vs. No). These analyses aimed to provide preliminary evidence of measurement stability within the target population. As shown in Table 7, configural invariance was established for the awareness of AI in healthcare, with acceptable model fit indices (*χ* ^2^/df = 1.914, RMSEA = 0.092, CFI = 0.938, TLI = 0.924, and SRMR = 0.068). Metric invariance was supported by the nonsignificant change in chi‐square (Δ*χ* ^2^ = 5.972, Δdf = 13, and *p* = 0.947) and the minimal changes in CFI (ΔCFI = 0.003) and RMSEA (ΔRMSEA = −0.006), both within the recommended thresholds of ΔCFI ≤ 0.010 and ΔRMSEA ≤ 0.015 [41, 44]. Scalar invariance was also achieved (Δ*χ* ^2^ = 13.005, Δdf = 13, *p* = 0.447, ΔCFI = 0.000, and ΔRMSEA = −0.003), indicating that both factor loadings and item intercepts were equivalent across groups with and without the awareness of AI in healthcare.

**TABLE 7:** Measurement invariance of the scale on the awareness of AI in healthcare.

Model	*χ* ^2^	df	*χ* ^2^ */*df	RMSEA (CI 90%)	CFI	TLI	SRMR	Δ*χ* ^2^	Δdf	*p*
Configural invariance	325.309	170	1.914	0.092 (0.076–0.107)	0.938	0.924	0.068			
Metric invariance	331.281	183	1.810	0.086 (0.071–0.101)	0.941	0.932	0.077	5.972	13	0.947
Scalar invariance	344.286	196	1.757	0.083 (0.069–0.098)	0.941	0.937	0.078	13.005	13	0.447

Similarly, as presented in Table 8, measurement invariance across the awareness of AI in nursing practice was supported. Configural invariance showed acceptable fit (*χ* ^2^/df = 2.041, RMSEA = 0.098, CFI = 0.929, TLI = 0.912, and SRMR = 0.063). Both metric invariance (Δ*χ* ^2^ = 7.112, Δdf = 13, *p* = 0.896, ΔCFI = 0.002, and ΔRMSEA = −0.005) and scalar invariance (Δ*χ* ^2^ = 6.916, Δdf = 13, *p* = 0.906, ΔCFI = 0.003, and ΔRMSEA = −0.005) were confirmed, demonstrating that the scale functions equivalently regardless of whether nurses are aware of AI applications in nursing.

**TABLE 8:** Measurement invariance of the scale on the awareness of AI in nursing practice.

Model	*χ* ^2^	df	*χ* ^2^ */*df	RMSEA (CI 90%)	CFI	TLI	SRMR	Δ*χ* ^2^	Δdf	*p*
Configural invariance	346.952	170	2.041	0.098 (0.083–0.112)	0.929	0.912	0.063			
Metric invariance	354.064	183	1.935	0.093 (0.078–0.107)	0.931	0.921	0.073	7.112	13	0.896
Scalar invariance	360.98	196	1.842	0.088 (0.074–0.102)	0.934	0.929	0.074	6.916	13	0.906

Table 9 displays the results for adoption of AI technologies in the hospital. Configural invariance was established with acceptable fit indices (*χ* ^2^/df = 2.074, RMSEA = 0.099, CFI = 0.928, TLI = 0.911, and SRMR = 0.061). Metric invariance was supported (Δ*χ* ^2^ = 14.053, Δdf = 13, *p* = 0.370, ΔCFI = 0.000, and ΔRMSEA = −0.003). Although the chi‐square difference test for scalar invariance reached statistical significance (Δ*χ* ^2^ = 31.412, Δdf = 13, and *p* = 0.003), the changes in CFI (ΔCFI = −0.008) and RMSEA (ΔRMSEA = 0.001) remained within the recommended cutoffs of ≤ 0.010 and ≤ 0.015, respectively. Given that the chi‐square test is highly sensitive to the sample size, the ΔCFI and ΔRMSEA criteria are considered more reliable indicators of invariance [41, 44]. Thus, scalar invariance was considered tenable across groups with different levels of hospital AI adoption.

**TABLE 9:** Measurement invariance of the scale on the adoption of AI technologies in the hospital.

Model	*χ* ^2^	df	*χ* ^2^ */*df	RMSEA (CI 90%)	CFI	TLI	SRMR	Δ*χ* ^2^	Δdf	*p*
Configural invariance	352.580	170	2.074	0.099 (0.085–0.114)	0.928	0.911	0.061			
Metric invariance	366.633	183	2.003	0.096 (0.082–0.110)	0.928	0.917	0.074	14.053	13	0.370
Scalar invariance	398.045	196	2.031	0.097 (0.083–0.111)	0.920	0.915	0.076	31.412	13	0.003

Taken together, these findings provide preliminary evidence that the Chinese version of ASUAITIN exhibits configural, metric, and scalar invariances across nurses with varying levels of AI awareness and exposure. This supports the scale’s stability and appropriateness for group comparisons within the Chinese nursing population.

## 4. Discussion

With the widespread application of AI technologies in the nursing field, scientifically evaluating nursing staff’s attitude toward the use of AI technologies has become a basic prerequisite for promoting the deep integration and promotion of AI technologies [9]. This study developed a Chinese version of ASUAITIN through a rigorous cross‐cultural adaptation process, including standardized translation and psychometric attribute assessment. The final version retained all 15 items of the original scale and demonstrated good internal consistency reliability. Validity testing, conducted through EFA, confirmed the expected two‐factor structure supporting the scale’s construct validity. The results indicate that the psychological structure of attitudes toward AI in the nursing field exhibits fundamental structural consistency across Eastern and Western professional contexts, while also revealing subtle cultural and linguistic differences in the expression of certain attitudes within the Chinese healthcare environment. Furthermore, the Chinese version of ASUAITIN is able to capture nurses’ situational attitudinal tendencies toward AI integration, including acceptance and concern. This capability provides a methodological basis for more accurately assessing readiness for AI applications, helping to identify training needs, and facilitating the design of targeted educational and supportive interventions to promote the active and effective application of AI technologies by nurses in their nursing practice.

The cross‐cultural adaptation of assessment tools is crucial to ensuring their semantic accuracy, conceptual equivalence, and cultural appropriateness when applied in new linguistic and professional contexts [46]. The main challenges in adapting tools like ASUAITIN lie not only in linguistic translation but also in ensuring that they conform to the cultural and professional norms of the target population while retaining their intended meaning [47, 48]. Based on Delphi expert feedback, no items were deleted, but some items underwent subtle yet meaningful wording modifications: for example, item 1 was changed from “I think AI technologies will be a hindrance to the application of nursing care practices” to “I think AI technologies are detrimental to the development of nursing work,” which softens the tone and better reflects an attitude assessment rather than a definitive prediction, a subtlety that aligns with Chinese professional communication styles. Similarly, item 11 was adjusted from “prediction” to “preassessment,” a term that better aligns with Chinese clinical nursing terminology, thus improving professional relevance and understanding. The results of the cross‐cultural adaptation phase of this study indicate that the Chinese version of ASUAITIN achieved satisfactory semantic and conceptual equivalence with the original scale. The expert review further confirmed that the scale comprehensively reflects both the positive and negative attitudinal dimensions, thereby supporting its content validity and suitability for use in clinical and managerial assessments. Additionally, feedback from the pretest with 15 clinical nurses confirmed that the items were clear, interpretable, and linguistically appropriate, further supporting the scale’s face validity and practical applicability.

The two‐factor structure—positive and negative attitudes—was clearly replicated in the Chinese sample, supporting the cross‐cultural stability of the original scale’s theoretical framework. This structure aligns with the TAM, which posit that perceived usefulness and ease of use foster positive attitudes, while concerns related to ethics, depersonalization, or professional autonomy contribute to negative attitudes [19, 20]. Notably, the two dimensions are not polar opposites but coexisting evaluative tendencies, a pattern consistent with dialectical thinking commonly observed in East Asian cultures [22]. This structural invariance across cultural contexts supports the universality of the attitude construct while acknowledging culturally shaped expressions. It is worth noting that the Chinese version of ASUAITIN evaluates nursing staff’s attitude toward the use of AI technologies from two dimensions: negative and positive. This is different from the structural dimension of the scale for evaluating nurses’ attitude toward the use of AI developed by Yıldırım and Karaman [49]. This study divides attitude assessment into four dimensions: nursing practice, organization, AI readiness, and ethics. This reflects the different emphases of different researchers in evaluating attitude toward AI technologies. The scale developed by Yıldırım and Karaman focuses on helping medical institutions identify nursing staff’s attitudes at different levels, while the Chinese version of the scale in this study is suitable for screening nursing staff’s overall attitude tendencies [49]. While the two‐factor structure was supported, it is worth noting that the separation between positive and negative attitudes aligns with the polarity of item wording, raising the possibility that a wording effect may partially contribute to the observed factor structure. While our comparative analysis against a one‐factor model provides evidence suggesting that this structure is not merely an artifact, the potential influence of item wording cannot be entirely ruled out. Future research employing bifactor models or correlated method factor approaches could help disentangle substantive construct variance from variance attributable to item wording.

The Chinese version of ASUAITIN has good internal consistency and high reliability. Therefore, the Chinese version of the scale can be used to assess Chinese nurses’ attitudes toward the use of AI technologies. It is worth noting that the overall Cronbach’s α coefficient of this study is slightly lower than that of the original study, which may be related to sample characteristics and cultural differences. The original study samples only came from three departments of internal medicine, surgery, and intensive care units in a hospital in English, while the samples of this study came from 20 different hospitals in Fujian Province, China, including different populations from multiple departments such as internal medicine, surgery, intensive care units, emergency, gynecology, and pediatrics. The cultural differences between the two regions may cause participants to have different understandings and responses to the scale items, resulting in increased discreteness of the item scores and reduced internal consistency. However, the overall Cronbach’s α coefficient of this study is still higher than 0.7, indicating that the data quality still has good reliability. In addition, in terms of data collection methods, this study used an online questionnaire to collect data, which has many advantages over the face‐to‐face data collection method of the original study. For example, online surveys can break through geographical restrictions to obtain a more representative large sample, are efficient and convenient, reduce economic costs, save time, ensure anonymity, and facilitate data entry and analysis [50, 51]. Previous studies have shown that Cronbach’s α coefficient is equivalent between online and offline data collection methods [52], and the response rate of 81.34% in this study is also significantly higher than the acceptable standard of 75% [53], further verifying the scientificity and feasibility of the online method.

Compared with the study by Yılmaz et al., we added items such as awareness of the use of AI in healthcare or nursing practice, frequency, and proficiency of the use of AI technologies in the sociodemographic survey to gain a more comprehensive understanding of the current status of AI technologies application among Chinese nursing staff [18]. In our survey, about half of the nurses were aware of the use of AI in healthcare or nursing practice, but their understanding, proficiency, and frequency of the use of AI technologies were not very satisfactory. In terms of understanding and proficiency, the total proportion of “very good” and “excellent” was only 8.49% and 2.98%, respectively. In terms of frequency, the proportion of frequent use (daily use) was only 8.49%, both lower than the study by Labrague et al. [54]. This may be related to the lack of systematic training on AI technologies provided by medical institutions. Although 66.74% of the participants said that their units had adopted AI technologies, the lack of relevant training plans would prevent AI technologies from being truly used among nurses [1]. In addition, nurses’ concerns and worries about the clinical application of AI technologies may affect their behavioral intention to use AI technologies in the future. In a cross‐sectional study of Chinese nursing students and nurses [9], 53% of the participants agreed that AI would replace the work of nurses and 95.7% of the participants believed that it was necessary to strengthen the medical ethics of nursing AI. The above data suggest that the promotion of AI technologies in the future needs to understand nurses’ attitudes toward their use and to specify targeted intervention measures or training courses based on the results of the survey to eliminate cognitive misunderstandings and ethical issues in the promotion of the technologies [55]. Since this study mainly focuses on the cross‐cultural adaptation and validation of ASUAITIN, future studies will further explore these influencing factors.

### 4.1. Limitation and Future Research Directions

There are some limitations to the study that deserve consideration and emphasis. First, although our analysis supports a two‐factor structure for the Chinese version of the ASUAITIN, it is worth noting that the distinction between positive and negative attitudes aligns with the polarity of the item wording. Future research should explore this issue by employing bifactor models or correlated method factor approaches to disentangle substantive variance from variance attributable to item wording. Second, regarding the structural validity of the Chinese version of ASUAITIN, the model fit indices demonstrated acceptable but not optimal levels. We acknowledge that while sufficient to support the scale’s structural validity in the Chinese nursing context, the model fit observed in this study still represents a limitation. Future research should aim to further optimize the scale’s structural properties by employing larger and more geographically diverse samples, and by exploring alternative models that explicitly account for potential wording effects. Third, the measurement invariance analyses in this study were conducted using grouping variables derived from single‐item, self‐reported demographic questions. While these measures were appropriate for exploratory subgroup comparisons in this initial validation study, they are not validated instruments and may introduce measurement error. Future research should replicate these invariance analyses using more robust, multi‐item instruments specifically designed to assess AI‐related knowledge, awareness, and exposure, which would provide more reliable evidence of the scale’s measurement stability across relevant subgroups. Fourth, this study did not conduct cross‐cultural measurement invariance testing between the Chinese sample and the original scale development sample. The absence of such invariance testing limits our ability to confidently assert that the Chinese version of ASUAITIN is directly comparable to the original scale. Future research should, therefore, aim to collect data from both Chinese and original‐scale populations to formally test cross‐cultural measurement invariance, which would provide stronger evidence for the scale’s applicability across diverse cultural settings. In addition, although we were very careful in translating and cross‐culturally adapting ASUAITIN, it is still possible that some cultural or linguistic nuances were overlooked. Considering these potential limitations is essential to maintain a cautious approach to interpreting the results and conducting follow‐up research related to ASUAITIN.

## 5. Implications for Nursing Management

The Chinese version of ASUAITIN in this study provides important research and practical guidance for the promotion and application of AI technologies in the nursing management. The scale comprehensively evaluates the attitude of nursing staff toward the use of AI technologies from two dimensions: positive and negative. Based on the assessment results, nursing managers and healthcare institutions may use the scale to identify attitudinal differences toward AI technologies and to develop more targeted training and implementation strategies. The use of this scale in further expanding the sample range and medical institution type survey will help establish a more representative norm of Chinese nurses’ AI attitudes, clarify the attitude baseline values of nurses in different levels of hospitals, clinical specialties, and age groups, and provide a reference standard for the precise implementation of AI technologies in the nursing field. At the same time, incorporating the evaluation of attitude toward the use of AI technologies into the evaluation indicators of the nurses’ continuing education system and the intelligent construction of hospitals will effectively promote the deep integration of nursing practice and AI technologies and ultimately promote the development of nursing services toward intelligence and humanity.

## 6. Conclusion

Based on the translation, cross‐cultural adaptation, and content validation of ASUAITIN, we concluded that the Chinese version of ASUAITIN has good reliability and validity in assessing the attitude toward the use of AI technologies in the Chinese nursing field. Our study provides data support for the use of AI in the global nursing field from a Chinese perspective. Based on the attitude level, it may facilitate AI technologies with high clinical nursing adaptability and design the targeted use training programs of AI technologies, thereby enhancing the intelligent development of the nursing field, ultimately promoting the precision and efficiency of the use of AI technologies in the nursing field.

**Nomenclature**
AI: Artificial intelligence
ASUAITIN: The Attitude Scale toward the Use of Artificial Intelligence Technologies in Nursing
AVE: Average variance extracted
CFA: Confirmatory factor analysis
CFI: Comparative fit index
CITC: The corrected item‐total correlation
EFA: Exploratory factor analysis
GAAIS: The General Attitudes Toward Artificial Intelligence Scale
I‐CVI: The content validity index at the item level
KMO: Kaiser–Meyer–Olkin test
PCA: Principal component analysis
RMSEA: Root mean square error of approximation
S‐CVI/Ave: The average content validity index at the scale level
SRMR: Standardized root mean square residual
TAM: Technology acceptance model
TLI: Tucker–Lewis goodness‐of‐fit index
*χ* ^2^ */*df: Chi‐square/degree of freedom

## Author Contributions

Zhi Chen and Xingxing Wu wrote the manuscript. Zhi Chen and Rong Lin helped with statistics. Rong Lin reviewed and edited, and Shufe Ke helped with data collection. Hong Li supervised the study. All authors certify that they have participated successfully in the work to take public responsibility for the content, including participation in the concept, design, analysis, writing, or revision of the manuscript. Zhi Chen and Xingxing Wu are the co‐first authors of this paper.

## Funding

This work was supported by the National Natural Science Foundation of China (grant number 72104050) and Fujian Province Science and Technology‐Economy Integration Service Platform (grant number FJKX‐2024XRH04).

## Disclosure

No artificial intelligence or language models were used in any aspect of this study, including research design, data collection, data analysis, or interpretation of findings.

## Ethics Statement

This study was approved by the Biomedical Research Ethics Review Committee of Fujian Medical University (approval number: 2025–307). Informed consent was obtained from all participants. This study was conducted in accordance with the principles outlined in the Declaration of Helsinki.

## Conflicts of Interest

The authors declare no conflicts of interest.

## Supporting Information

Additional supporting information can be found online in the Supporting Information section.

## Supporting information


**Supporting Information 1** This supporting information 1 provides an English version of online questionnaire and a Chinese version of online questionnaire, which include a demographic questionnaire and the Attitude Scale toward the Use of Artificial Intelligence Technologies in Nursing (ASUAITIN), respectively.

**Supporting Information 2** This supporting information 2 provides the details of the Delphi consultation.

## Data Availability

The data that support the findings of this study are available on request from the corresponding authors. The data are not publicly available due to privacy or ethical restrictions.
