# Supplementary material for: Measuring Positive and Negative Attitudes Associated With the Use of Artificial Intelligence in Nursing Profession: Cross‐Cultural Adaptation and Psychometric Analysis of ASUAITIN
Source: J Nurs Manag. 2026 Apr 29;2026:5888837. doi: 10.1155/jonm/5888837 (PMC13126244; doi:10.1155/jonm/5888837)
Supplement: Supplementary file 1 — Supporting Information 1 This supporting information 1 provides an English version of online questionnaire and a Chinese version of online questionnaire, which include a demographic questionnaire and the Attitude Scale toward the Use of Artificial Intelligence Technologies in Nursing (ASUAITIN), respectively. [file JONM-2026-5888837-s002.docx]

**Supplementary material 1**

This is complementary to the article entitled “Measuring Positive and Negative Attitudes Associated With the Use of Artificial Intelligence in Nursing Profession: Cross-Cultural Adaptation and Psychometric Analysis of ASUAITIN”. This supplementary material provides the English version of online, which include a demographic questionnaire, an English version of the original Attitude Scale towards the Use of Artificial Intelligence Technologies in Nursing (ASUAITIN) and a Chinese version of the Attitude Scale towards the Use of Artificial Intelligence Technologies in Nursing (ASUAITIN).

**English version of online questionnaire**

**Demographic questionnaire**

1. Gender:

☐Male

☐Female

2. Age: years old

3. Work experience: years

4. Education level:

☐College degree or below

☐Bachelor degree

☐Master degree or above

5. Marital status

☐Single

☐Married

☐Other

6. Job title

☐Nurse

☐Senior nurse

☐Senior nurse in charge

☐Associate chief senior nurse and above

7. Hospital department

☐Internal medicine

☐Surgery

☐Emergency

☐Intensive care unit

☐Other

8. Weekly working hours

☐Less than 41 hours

☐More than 39 hours

9. Do you understand the use of artificial intelligence technologies in the healthcare?

☐Yes

☐No

10. Do you understand the use of artificial intelligence technologies in nursing?

☐Yes

☐No

1. How well do you understand artificial intelligence technologies?

☐Poor

☐Average

☐Good

☐Very good

☐Excellent

1. How often do you use artificial intelligence technologies?

☐ Frequent (Daily use)

☐ Regular (Weekly use)

☐ Occasionally (Monthly/Yearly use)

☐ Never

1. How proficient are you in artificial intelligence technologies?

☐Poor

☐Below average

☐Average

☐Above average

☐Excellent

1. Has your hospital/unit adopted artificial intelligence technologies?

☐Yes

☐No

**English version of the original Attitude Scale towards the Use of Artificial Intelligence Technologies in Nursing (ASUAITIN)**

1. I think artificial intelligence technologies will be a hindrance to the application of nursing care practices.

☐Completely disagree

☐Disagree

☐Unsure

☐Agree

☐Completely agree

1. I feel uncomfortable when I think how artificial intelligence technologies will be used in the future in nursing care.

☐Completely disagree

☐Disagree

☐Unsure

☐Agree

☐Completely agree

1. I think that the nursing profession will be harmed if artificial intelligence technologies are used more in the future.

☐Completely disagree

☐Disagree

☐Unsure

☐Agree

☐Completely agree

1. I think that the use of artificial intelligence technologies in nursing care will put patient safety at risk.

☐Completely disagree

☐Disagree

☐Unsure

☐Agree

☐Completely agree

1. I think it isn’t right to use artificial intelligence technologies in nursing.

☐Completely disagree

☐Disagree

☐Unsure

☐Agree

☐Completely agree

1. I think that the use of artificial intelligence technologies in nursing practice can cause ethical problems.

☐Completely disagree

☐Disagree

☐Unsure

☐Agree

☐Completely agree

1. Artificial intelligence technologies can provide new opportunities for nurses.

☐Completely disagree

☐Disagree

☐Unsure

☐Agree

☐Completely agree

1. The field of use of artificial intelligence technologies in nursing is wide.

☐Completely disagree

☐Disagree

☐Unsure

☐Agree

☐Completely agree

1. There are many beneficial applications of artificial intelligence technologies in nursing.

☐Completely disagree

☐Disagree

☐Unsure

☐Agree

☐Completely agree

1. I would prefer to use a system with artificial intelligence for some routine nursing procedures, like vital findings and pain assessment.

☐Completely disagree

☐Disagree

☐Unsure

☐Agree

☐Completely agree

1. Predictions about the patient by artificial intelligence technologies (nursing diagnosis, care needs, etc.) can provide practical opportunities to make the profession easier.

☐Completely disagree

☐Disagree

☐Unsure

☐Agree

☐Completely agree

1. I think that benefitting from artificial intelligence technologies in nursing is achievable.

☐Completely disagree

☐Disagree

☐Unsure

☐Agree

☐Completely agree

1. I would like to have skills in learning and using artificial intelligence technologies in nursing.

☐Completely disagree

☐Disagree

☐Unsure

☐Agree

☐Completely agree

1. I would like to have training on learning the use of artificial intelligence technologies in nursing.

☐Completely disagree

☐Disagree

☐Unsure

☐Agree

☐Completely agree

1. I think it is necessary to include artificial intelligence technologies in the core curriculum of nursing education.

☐Completely disagree

☐Disagree

☐Unsure

☐Agree

☐Completely agree

**Chinese version of the Attitude Scale towards the Use of Artificial Intelligence Technologies in Nursing (ASUAITIN)**

1. I believe that artificial intelligence technologies are not conducive to the delivery of nursing care.

☐Completely disagree

☐Disagree

☐Unsure

☐Agree

☐Completely agree

1. When I think about the future application of artificial intelligence technologies in nursing services, I feel uncomfortable.

☐Completely disagree

☐Disagree

☐Unsure

☐Agree

☐Completely agree

1. I believe that the increased use of artificial intelligence technologies in the future will be detrimental to the nursing profession.

☐Completely disagree

☐Disagree

☐Unsure

☐Agree

☐Completely agree

1. I believe that the use of artificial intelligence technologies in nursing services may pose risks to patient safety.

☐Completely disagree

☐Disagree

☐Unsure

☐Agree

☐Completely agree

1. I believe that the use of artificial intelligence technologies in the field of nursing is inappropriate.

☐Completely disagree

☐Disagree

☐Unsure

☐Agree

☐Completely agree

1. I believe that the use of artificial intelligence technologies in nursing practice may raise ethical concerns.

☐Completely disagree

☐Disagree

☐Unsure

☐Agree

☐Completely agree

1. Artificial intelligence technologies can provide new opportunities for nurses.

☐Completely disagree

☐Disagree

☐Unsure

☐Agree

☐Completely agree

1. Artificial intelligence technologies have a wide range of applications in nursing.

☐Completely disagree

☐Disagree

☐Unsure

☐Agree

☐Completely agree

1. Artificial intelligence technologies have many beneficial applications in the field of nursing.

☐Completely disagree

☐Disagree

☐Unsure

☐Agree

☐Completely agree

1. I would prefer to use artificial intelligence systems to perform certain routine nursing tasks, such as vital signs monitoring and pain assessment.

☐Completely disagree

☐Disagree

☐Unsure

☐Agree

☐Completely agree

1. Artificial intelligence technologies can provide practical support for patient pre-assessment (e.g., nursing diagnosis and identification of nursing needs), thereby facilitating nursing practice and reducing workload.

☐Completely disagree

☐Disagree

☐Unsure

☐Agree

☐Completely agree

1. I believe that it is feasible to achieve benefits from the use of artificial intelligence technologies in the field of nursing.

☐Completely disagree

☐Disagree

☐Unsure

☐Agree

☐Completely agree

1. I would like to acquire the skills needed to learn and use artificial intelligence technologies in the field of nursing.

☐Completely disagree

☐Disagree

☐Unsure

☐Agree

☐Completely agree

1. I would like to receive training on the application of artificial intelligence technologies in the field of nursing.

☐Completely disagree

☐Disagree

☐Unsure

☐Agree

☐Completely agree

1. I believe it is necessary to incorporate artificial intelligence technologies into the core curriculum of nursing education.

☐Completely disagree

☐Disagree

☐Unsure

☐Agree

☐Completely agree
