# Supplementary material for: Measuring Positive and Negative Attitudes Associated With the Use of Artificial Intelligence in Nursing Profession: Cross‐Cultural Adaptation and Psychometric Analysis of ASUAITIN
Source: J Nurs Manag. 2026 Apr 29;2026:5888837. doi: 10.1155/jonm/5888837 (PMC13126244; doi:10.1155/jonm/5888837)
Supplement: Supplementary file 2 — Supporting Information 2 This supporting information 2 provides the details of the Delphi consultation. [file JONM-2026-5888837-s001.docx]

**Supplementary material 2**

This is complementary to the article entitled “Measuring Positive and Negative Attitudes Associated With the Use of Artificial Intelligence in Nursing Profession: Cross-Cultural Adaptation and Psychometric Analysis of ASUAITIN”. This supplementary material provides the details of the Delphi consultation.

**Table S1. Basic information of the expert panel for cross-cultural adaption**

| **Number** | **Age (years)** | **Professional Title** | **Years of Research/Work Experience** | **Educational Background** | **Research Field** |
| --- | --- | --- | --- | --- | --- |
| N1 | 56 | Professor | 31 | PhD | Clinical Nursing |
| N2 | 42 | Associate Chief Nurse | 17 | PhD | Nursing Education |
| N3 | 46 | Chief Nurse | 23 | PhD | Clinical Nursing |
| N4 | 38 | Nurse-in-Charge | 15 | Master's | Clinical Nursing |
| N5 | 35 | Nurse-in-Charge | 12 | Master's | Nursing Informatics |
| N6 | 30 | Nurse-in-Charge | 7 | PhD | Clinical Nursing |
| N7 | 35 | Lecturer | 12 | PhD | Nursing Informatics |
| N8 | 30 | Teaching Assistant | 5 | Master's | Nursing Education |
| N9 | 39 | Associate Professor | 7 | PhD | Nursing Education |
| N10 | 26 | Nurse-in-Charge | 5 | Master's | Nursing Management |
| N11 | 45 | Chief Nurse | 23 | Master's | Nursing Management |

**Table S2. Summary of Item Revision Comments and Expert Scores**

| **Item** | **Original English Text** | **Translated Item** | **Comments (Organized by Expert)** | **Expert Rating**  **(Linguistic Consistency / Cultural Relevance)** |
| --- | --- | --- | --- | --- |
| **Title** | Attitude Scale towards the Use of Artificial Intelligence Technologies in Nursing | Attitude Scale towards the Use of Artificial Intelligence Technologies in Nursing | N1: None N2: Can "Technologies" be deleted? N3: None N4: None N5: None N6: None N7: None N8: None N9: None N10: None N11: None | N1: 4/4 N2: 4/4 N3: 3/4 N4: 4/4 N5: 4/4 N6: 4/4 N7: 4/4 N8: 4/4 N9: 4/4 N10: 4/4 N11: 4/4 |
| **Dimension 1 (Negative)** | a negative attitude to AI technologies in nursing practice | A negative attitude towards AI technologies in nursing practice | N1: None N2: None N3: None N4: A negative attitude towards AI technologies in nursing practice N5: A negative attitude towards using AI technologies in nursing practice N6: None N7: A negative attitude towards using AI technologies in nursing practice N8: None N9: None N10: None N11: None | N1: 4/4 N2: 4/4 N3: 3/4 N4: Not rated N5: 4/4 N6: 4/4 N7: 4/4 N8: 4/4 N9: 4/4 N10: 4/4 N11: 4/4 |
| **1** | I think artificial intelligence technologies will be a hindrance to the application of nursing care practices. | I think AI technologies will hinder the application of nursing care practices. | N1: None N2: This phrase "nursing care practices" is unclear. The sentence is not clearly expressed. N3: None N4: I think AI technologies will hinder the application of nursing practices. N5: Suggest changing to "hinder nursing work" to be closer to clinical practice. N6: Would "hinder" be better than "hindrance"? Hindering development / hindering application. N7: Nursing care practices N8: None N9: Nursing care practices N10: If the target users are clinical frontline nurses and this is a self-assessment scale, translating "nursing practices" to "nursing work" would be more down-to-earth and easier to understand. "Nursing practice" includes procedures like operations, assessments, diagnoses, and care planning. Replacing it with "nursing work" feels easy to understand. Also, "the application of nursing care practices" sounds a bit awkward. The term "hindrance" is very strong and suggestive. Could we consider translating this sentence as: "I think AI technologies are not conducive to / will hinder the implementation/carrying out of nursing work." N11: None | N1: 4/4 N2: 4/4 N3: 4/4 N4: 4/4 N5: 4/4 N6: 4/4 N7: 4/4 N8: 4/4 N9: 3/4 N10: 3/3 N11: 4/4 |
| **2** | I feel uncomfortable when I think how artificial intelligence technologies will be used in the future in nursing care. | I feel uneasy when I think about how AI technologies will be used in nursing care in the future. | N1: None N2: Can "how" be deleted? N3: None N4: None N5: Suggest changing to "nursing care". N6: Would "uncomfortable" be more suitable? N7: Can "how" be deleted? N8: None N9: Nursing care N10: None N11: None | N1: 4/4 N2: 4/4 N3: 4/4 N4: 4/4 N5: 4/4 N6: 3/4 N7: 4/4 N8: 4/4 N9: 3/4 N10: 4/4 N11: 4/4 |
| **3** | I think that the nursing profession will be harmed if artificial intelligence technologies are used more in the future. | I think the nursing profession will be harmed if AI technologies are used more in the future. | N1: None N2: Change "used" to "applied"? N3: None N4: None N5: Suggest changing to "nursing profession" (using term for industry). N6: None N7: The context of "nursing profession" leans more towards education, suggest changing to "nursing field" or "nursing career". N8: None N9: None N10: The context of "nursing profession" is more easily associated with education and universities. If the scale's target users are nurses, could "profession" be translated as "field" or "career"? "Profession" itself carries these meanings. N11: None | N1: 4/4 N2: 4/4 N3: 3/3 N4: 4/4 N5: 4/4 N6: 4/4 N7: 4/4 N8: 4/4 N9: 4/4 N10: 3/4 N11: 4/4 |
| **4** | I think that the use of artificial intelligence technologies in nursing care will put patient safety at risk. | I think using AI technologies in nursing care will put patient safety at risk. | N1: None N2: None N3: Suggest changing "in nursing" to "in nursing practice" N4: I think using AI technologies in nursing will put patient safety at risk. N5: Suggest changing to "in nursing practice". N6: None N7: Suggest changing to "will put patient safety at risk". N8: None N9: None N10: None N11: None | N1: 4/4 N2: 4/4 N3: 3/4 N4: 4/3 N5: 4/4 N6: 4/4 N7: 4/4 N8: 4/4 N9: 4/4 N10: 4/4 N11: 4/4 |
| **5** | I think it isn't right to use artificial intelligence technologies in nursing. | I think it is inappropriate to use AI technologies in nursing. | N1: None N2: "Right" doesn't seem clearly expressed here. N3: None N4: I think it is inappropriate to use AI technologies in nursing. N5: "Right" is too strong, suggest changing to "appropriate". N6: None N7: "Right" doesn't seem clearly expressed here. N8: None N9: None N10: Scales need to avoid strongly suggestive words. Inappropriate -> not right / unsuitable? N11: None | N1: 4/4 N2: 4/4 N3: 4/4 N4: 4/3 N5: 4/4 N6: 4/4 N7: 4/4 N8: 4/4 N9: 4/4 N10: 3/3 N11: 4/4 |
| **6** | I think that the use of artificial intelligence technologies in nursing practice can cause ethical problems. | I think using AI technologies in nursing practice can cause ethical problems. | N1: None N2: None N3: None N4: None N5: Remove "can". N6: None N7: Suggest consistently using "nursing practice" or "nursing work". N8: None N9: Remove "can" N10: If we change it to "nursing work" above, then unify it. If not, use "nursing practice". N11: None | N1: 4/4 N2: 4/4 N3: 4/4 N4: 4/4 N5: 4/4 N6: 4/4 N7: 4/4 N8: 4/4 N9: 3/4 N10: 4/4 N11: 4/4 |
| **Dimension 2 (Positive)** | a positive attitude by nurses to AI technologies in nursing practice | A positive attitude of nurses towards AI technologies in nursing practice | N1: None N2: None N3: None N4: None N5: A positive attitude towards using AI technologies in nursing practice N6: A positive attitude of nurses towards AI technologies in nursing practice N7: A positive attitude towards using AI technologies in nursing practice N8: None N9: None N10: None N11: A positive attitude of nurses towards AI technologies in nursing practice | N1: 4/4 N2: 4/4 N3: 3/4 N4: 4/4 N5: 4/4 N6: 4/4 N7: 4/4 N8: 4/4 N9: 4/4 N10: 4/4 N11: 3/4 |
| **7** | Artificial intelligence technologies can provide new opportunities for nurses. | AI technologies can provide new opportunities for nurses. | N1: None N2: None N3: None N4: None N5: None N6: None N7: Suggest changing to "opportunities". N8: None N9: None N10: None N11: None | N1: 4/4 N2: 4/4 N3: 4/4 N4: 4/4 N5: 4/4 N6: 4/4 N7: 4/4 N8: 4/4 N9: 4/4 N10: 4/4 N11: 4/4 |
| **8** | The field of use of artificial intelligence technologies in nursing is wide. | The application field of AI technologies in nursing is very broad. | N1: None N2: None N3: The phrase "application field in nursing" is not objectionable, but it's a bit awkward. Please reconsider. N4: None N5: Suggest changing to "scope of application is broad". N6: Suggest changing to "The field of application of AI technologies in nursing is broad." N7: None N8: None N9: None N10: None N11: The scope of application of AI technologies in the nursing field is broad. | N1: 4/4 N2: 4/4 N3: 3/4 N4: 4/4 N5: 4/4 N6: 3/4 N7: 4/4 N8: 4/4 N9: 4/4 N10: 4/4 N11: 3/3 |
| **9** | There are many beneficial applications of artificial intelligence technologies in nursing. | There are many beneficial applications of AI technologies in nursing. | N1: None N2: None N3: "There are many beneficial applications" is relatively awkward. Please reconsider. N4: None N5: The phrasing is awkward. Suggest changing to "There are many beneficial applications of AI technologies in the nursing field." N6: None N7: None N8: None N9: None N10: None N11: There are many beneficial applications of AI technologies in the nursing field. | N1: 4/4 N2: 4/4 N3: 4/3 N4: 4/4 N5: 4/4 N6: 4/4 N7: 4/4 N8: 4/4 N9: 4/4 N10: 4/4 N11: 4/3 |
| **10** | I would prefer to use a system with artificial intelligence for some routine nursing procedures, like vital findings and pain assessment. | I would prefer to use an AI system for some routine nursing procedures, such as vital signs monitoring and pain assessment. | N1: None N2: None N3: None N4: None N5: Routine nursing procedures -> routine nursing tasks/work. N6: None N7: Suggest changing to "routine nursing work". N8: None N9: None N10: Routine nursing procedures -> routine nursing tasks/work. Closer to clinical terminology. N11: I would prefer to use an AI system for some routine nursing tasks, such as vital signs monitoring and pain assessment. | N1: 4/4 N2: 4/4 N3: 4/4 N4: 4/4 N5: 4/4 N6: 4/4 N7: 4/4 N8: 4/4 N9: 4/4 N10: 4/3 N11: 3/3 |
| **11** | Predictions about the patient by artificial intelligence technologies (nursing diagnosis, care needs, etc.) can provide practical opportunities to make the profession easier. | Predictions about patients made by AI technologies (e.g., nursing diagnosis, care needs) can provide practical assistance to nursing work, making it easier. | N1: None N2: None N3: None N4: None N5: Suggest changing "predictions" to "pre-assessments". N6: None N7: None N8: None N9: None N10: None N11: None | N1: 4/4 N2: 4/4 N3: 4/4 N4: 4/4 N5: 4/4 N6: 4/3 N7: 4/4 N8: 4/4 N9: 4/4 N10: 4/4 N11: 4/4 |
| **12** | I think that benefitting from artificial intelligence technologies in nursing is achievable. | I believe that benefiting from AI technologies in nursing work is achievable. | N1: None N2: None N3: None N4: I think benefiting from AI technologies in nursing work is achievable. N5: Suggest deleting "and" to better emphasize "benefiting from". N6: None N7: None N8: None N9: None N10: This item has two meanings: one is that using AI technologies in nursing work is feasible, the other is that benefiting from using the technology is feasible. However, the original item seems to want to express the second meaning. So, should the translated item consider deleting "and"? N11: I believe that applying AI technologies in the nursing field and obtaining benefits from them is achievable. | N1: 4/4 N2: 4/4 N3: 4/4 N4: 3/4 N5: 4/4 N6: 4/4 N7: 4/4 N8: 4/4 N9: 4/4 N10: 4/4 N11: 4/3 |

**Table S3. Comparison of revisions to the items of the Attitude Scale towards the use of Artificial Intelligence Technologies in Nursing before and after the Delphi Expert Consultation.**

| **Item** | **Original Item in the Delphi Consultation Scale** | **Revised Item in the Final Scale** | **Explanation of Inconsistencies/Revisions** |
| --- | --- | --- | --- |
| 1 | I think AI technologies will hinder the application of nursing care practices. | I think AI technologies are not conducive to the implementation of nursing work. | The phrasing is more concise, changing from "hinder the application of nursing care practices" to "are not conducive to the implementation of nursing work." |
| 2 | I feel uneasy when I think about how AI technologies will be used in nursing care in the future. | I feel uncomfortable when I think about AI technologies being used in nursing services in the future. | Changed "uneasy" to "uncomfortable"; changed "how AI technologies will be used in nursing care" to "AI technologies being used in nursing services." |
| 3 | I think the nursing profession will be harmed if AI technologies are used more in the future. | I think the increased use of AI technologies in the future will lead to damage to the nursing field. | Slight structural adjustment, but the core meaning remains consistent. |
| 4 | I think using AI technologies in nursing care will put patient safety at risk. | I think using AI technologies in nursing services will put patient safety at risk. | Changed "endanger" to "put at risk" and added the word "services." |
| 5 | I think it is inappropriate to use AI technologies in nursing. | I think it is unsuitable to use AI technologies in the nursing field. | Changed "inappropriate" to "unsuitable," emphasizing applicability rather than right or wrong. |
| 6 | I think using AI technologies in nursing practice can cause ethical problems. | I think using AI technologies in nursing work will cause ethical problems. | Changed "nursing practice" to "nursing work" and made the tone more affirmative. |
| 7 | AI technologies can provide new opportunities for nurses. | AI technologies can provide new opportunities for nurses. | Changed "opportunities" to "opportunities" for more formal wording. |
| 8 | The application field of AI technologies in nursing is very broad. | The application field of AI technologies in nursing is broad. | Slight adjustment in tone, meaning unchanged. |
| 9 | There are many beneficial applications of AI technologies in nursing. | There are many beneficial applications of AI technologies in the nursing field. | Added the word "field" for greater clarity. |
| 10 | I would prefer to use an AI system for some routine nursing procedures, such as vital signs monitoring and pain assessment. | I would prefer to use an AI system for some routine nursing work, such as vital signs monitoring and pain assessment. | Changed "nursing procedures" to "nursing work" to better align with Chinese expression habits. |
| 11 | Predictions about patients made by AI technologies (e.g., nursing diagnosis, care needs) can provide practical assistance to nursing work, making it easier. | Pre-assessments of patients made by AI technologies (e.g., nursing diagnosis, care needs) can provide practical assistance to nursing work, making it easier. | Changed "predictions" to "pre-assessments," which is more accurate and aligns better with nursing terminology. |
| 12 | I believe that benefiting from AI technologies in nursing work is achievable. | I believe that benefiting from AI technologies in the nursing field is achievable. | Simplified the sentence structure for smoother expression. |
| 13 | I would like to acquire skills in learning and using AI technologies in nursing. | I would like to acquire skills in learning and using AI technologies in the nursing field. | Added "in the nursing field" for greater specificity. |
| 14 | I would like to receive training on learning how to use AI technologies in nursing work. | I would like to receive training on applying AI technologies in the nursing field. | The sentence is more concise, directly expressing the desire for training. |
| 15 | I think it is necessary to include AI technologies in the core curriculum of nursing education. | I think it is necessary to include AI technologies in the core curriculum of nursing education. | Content is consistent; no modifications were made. |
